# Sarcopenia and Survival in Patients With Head and Neck Squamous Cell Carcinoma Receiving Immune Checkpoint Inhibitors: A Systematic Review and Meta‐Analysis of Prognostic Association

**DOI:** 10.1002/cnr2.70695

**Published:** 2026-09-21

**Authors:** Diwakar Koirala, Nakendra Malla, Bivek Mishra, Ramesh Sapkota, Sahil Niraula, Kushal Bastola, Nirman Khatiwada, Manish Gahatraj, Rijan Kafle

**Affiliations:** ^1^ B.P. Koirala Institute of Health Sciences Dharan Nepal

**Keywords:** body composition, head and neck neoplasms, immune checkpoint inhibitors, meta‐analysis, prognosis, sarcopenia, skeletal muscle index, squamous cell carcinoma

## Abstract

**Background:**

Sarcopenia, characterized by loss of skeletal muscle mass and function, has emerged as a potential prognostic biomarker in oncology. Its role in patients with head and neck squamous cell carcinoma (HNSCC) treated with immune checkpoint inhibitors (ICIs) remains inadequately defined.

**Methods:**

A systematic review and meta‐analysis was conducted in accordance with PRISMA 2020. PubMed, Scopus, and the Cochrane Library were searched from inception to March 2026. Observational studies reporting baseline computed tomography (CT)‐defined sarcopenia, based on a dichotomized skeletal muscle index (SMI) or skeletal muscle area (SMA), in adults with HNSCC receiving ICIs were included. Studies using non‐radiological assessment, continuous‐only muscle indices, exposure definitions incorporating on‐treatment change, or populations restricted to progression after ICI therapy were excluded, and cohorts were screened for patient overlap. Hazard ratios (HRs) for overall survival (OS) and progression‐free survival (PFS) were pooled using a random‐effects model with the Hartung–Knapp–Sidik–Jonkman (HKSJ) method incorporating the truncated variance correction. Risk of bias was assessed with the QUIPS tool and certainty of evidence with GRADE as adapted for prognostic factor reviews.

**Results:**

Three retrospective cohort studies (354 patients) met the eligibility criteria. Under the pre‐specified primary estimator the pooled hazard ratio for OS was 2.05 (95% CI 1.00–4.20), an interval that marginally includes the null (common‐effect HR 2.05, 95% CI 1.48–2.84; *I* ^2^ = 0%); for PFS it was 1.85 (95% CI 1.05–3.27; *I* ^2^ = 0%), excluding the null only narrowly. The wide HKSJ intervals reflect the small number of studies. The pooled OS estimate is closely concordant with an independent published synthesis of the same question (HR 2.05). Certainty of evidence was rated low for both outcomes.

**Conclusion:**

Baseline CT‐defined sarcopenia was associated with an approximately two‐fold increase in the hazard of death, and of progression or death, in patients with HNSCC treated with ICIs. The primary OS analysis did not reach conventional statistical significance: under the pre‐specified HKSJ estimator its 95% confidence interval marginally included the null, and the PFS interval excluded the null only narrowly. The association therefore rests on the size and consistency of the point estimates, the absence of heterogeneity, the common‐effect interval and concordance with an independent published synthesis, rather than on the primary interval alone. The evidence base comprises three retrospective cohorts and certainty is low. Sarcopenia should be regarded as a prognostic rather than a predictive marker in this setting and must not be used to justify withholding immunotherapy. Prospective studies with standardized CT‐based definitions are required.

## 1 Introduction

Head and neck squamous cell carcinoma (HNSCC) comprises a distinct group of tumors that affect essential functions of the upper aerodigestive tract [1]. Head and neck cancers accounted for approximately ~4.5% of global cancer incidence and ~4.7% of cancer‐related mortality in 2022, corresponding to ~0.9 million new cases and ~0.46 million deaths worldwide [2]. Lip and oral cavity cancer accounts for ~2.0% of global incidence and ~1.9% of mortality, followed by laryngeal (~0.9% and ~1.1%), nasopharyngeal (~0.6% and ~0.8%), oropharyngeal (~0.5% and ~0.5%), and hypopharyngeal cancers (~0.4% and ~0.4%) [2]. Due to the aggressive nature of treatment, patients frequently experience multiple therapy‐related toxicities in the effort to achieve disease control [1]. Currently, surgery, radiotherapy, systemic therapy, or their combination constitute the standard of care in the management of head and neck cancer [3]. In recent years, immunotherapy, particularly checkpoint inhibitors, has emerged as a key therapeutic pillar in the management of multiple human malignancies [4]. Nivolumab and pembrolizumab are monoclonal antibodies targeting programmed death‐1 (PD‐1); initially approved by the Food and Drug Administration for metastatic melanoma in 2011, they are now used as first‐line (pembrolizumab) or later‐line (nivolumab) palliative treatment options in head and neck cancer [5].

Sarcopenia is characterized by a decline in muscle mass and function and is a major contributor to impaired wound healing and adverse outcomes in cancer patients [1]. Sarcopenia may not only affect treatment‐related toxicity but is also linked to poorer survival outcomes in this patient population [6]. In patients with advanced head and neck cancer, pre‐treatment sarcopenia assessed on routine cervical CT imaging may serve as a practical and objective surrogate marker of frailty, with potential utility in guiding treatment selection [6].

However, despite emerging evidence, the impact of sarcopenia on outcomes in patients with HNSCC, particularly in the context of immunotherapy, remains inadequately characterized; therefore, we conducted a systematic review and meta‐analysis to comprehensively evaluate its prognostic significance in this population.

## 2 Methods

### 2.1 Study Design and Reporting

This systematic review and meta‐analysis was conducted and reported in accordance with the Preferred Reporting Items for Systematic Reviews and Meta‐Analyses (PRISMA) 2020 statement [7]. The completed PRISMA 2020 checklist is provided in Supporting Information. A predefined protocol was registered in PROSPERO (registration number CRD420261334507; date of registration 13 Mar 2026 03:11 UTC; available at https://www.crd.york.ac.uk/PROSPERO/view/CRD420261334507). The registered record specifies baseline sarcopenia defined on computed tomography as the exposure of interest, which is the criterion enforced in the present analysis. Deviations from the registered protocol are declared below.

### 2.2 Protocol Deviations

The registered protocol specified baseline CT‐defined sarcopenia as the exposure of interest. In the originally submitted analysis, this criterion was applied inconsistently. During revision, every extracted estimate was independently re‐verified against its source publication; the eligibility criterion was enforced as registered, and four studies were consequently excluded. No other deviations from the registered protocol occurred.

### 2.3 Eligibility Criteria

Studies were selected using the population, exposure, comparators, outcomes, and study design (PECOS) framework appropriate for prognostic factor reviews.

Adult patients (≥ 18 years) with head and neck squamous cell carcinoma (HNSCC) receiving immune checkpoint inhibitor (ICI)‐based therapy (PD‐1, PD‐L1, or CTLA‐4 inhibitors) were eligible. The exposure of interest was baseline sarcopenia defined on computed tomography (CT) using the skeletal muscle index (SMI) or skeletal muscle area (SMA), dichotomized according to study‐specific thresholds; the anatomical level (e.g., L3, or a cervical [C3]‐based estimate of lumbar SMI) was recorded. Comparators were non‐sarcopenic patients as defined by the individual studies.

Eligible study designs were observational prognostic studies (retrospective or prospective cohorts). Studies were excluded if they were case reports or case series with fewer than 10 patients, reviews, editorials, conference abstracts without sufficient data, non‐human studies, or mixed cancer populations without extractable HNSCC‐specific data. In addition, the following exposure‐related exclusions were applied: (i) sarcopenia assessed by non‐radiological methods (e.g., bioelectrical impedance analysis); (ii) muscle status reported only as a continuous index without a dichotomous sarcopenia definition; (iii) exposure definitions incorporating on‐treatment change (e.g., cachexia combined with early weight loss during therapy); and (iv) cohorts restricted to patients who had already progressed after ICI therapy. Where multiple reports appeared to derive from overlapping cohorts (shared institutions, investigators, and accrual periods), only the cohort meeting the exposure definition with the most complete data was eligible for inclusion.

### 2.4 Search Strategy

A literature search was conducted in PubMed (NCBI web interface), Scopus (Elsevier), and the Cochrane Library (Wiley) from database inception to March 2026; all three databases were last searched in March 2026. The strategies were not identical across databases, and the strings as run are reproduced in Data S1. Only the Scopus strategy combined all three concepts of the review question: head and neck cancer, sarcopenia/body composition, and immune checkpoint inhibitors, within the TITLE‐ABS‐KEY field. The PubMed and Cochrane Library strategies combined two concepts only: head and neck cancer and sarcopenia/body composition; neither contained an immune checkpoint inhibitor concept, so the intervention restriction was applied at the screening stage rather than within the search string. Subsite terms (oral cavity, oropharyngeal, laryngeal and hypopharyngeal) were included in the Scopus and Cochrane Library strategies but not in the PubMed strategy, which used three population synonyms only. The Cochrane Library entry in Data S1 also records a free‐text phrase line run alongside the structured strategy. All strategies used free‐text terms; no controlled vocabulary (MeSH or Emtree) was applied in any database, and no filters or date, language, or publication‐type limits were used. Embase and Web of Science were not searched. The consequences of these limitations for the completeness of the evidence base are addressed in the Discussion.

**FIGURE 1:**
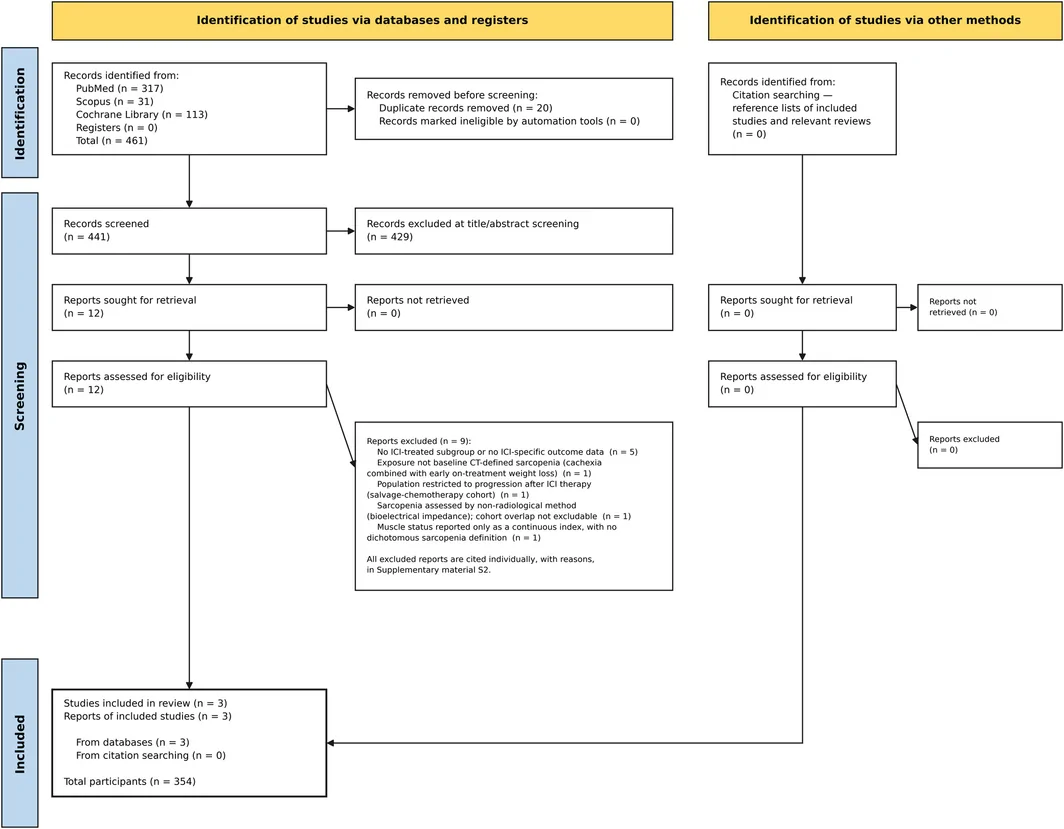
PRISMA flow diagram illustrating the study selection process.

The search yielded *n* = 317 records from PubMed, *n* = 31 from Scopus and *n* = 113 from the Cochrane Library, giving *n* = 461 records in total; after removal of *n* = 20 duplicates, *n* = 441 unique records were screened. These counts correspond to those reported in Figure 1.

Reference lists of included studies and relevant reviews were manually screened to identify additional eligible studies.

### 2.5 Study Selection

All identified records were imported into reference management software and duplicates were removed. Two reviewers independently screened titles and abstracts, followed by full‐text assessment of potentially eligible studies. Disagreements were resolved through discussion, with consultation from a third reviewer if required. Reports were additionally screened for cohort overlap by cross‐checking author lists, institutional affiliations, and accrual periods; where overlap was suspected, the source cohorts were compared and only one report per cohort was retained.

A total of 12 reports were assessed in full text, of which 3 studies were included in the final analysis (Figure 1). Full‐text reports excluded at this stage are cited, with itemized reasons for exclusion, in Data S2.

### 2.6 Data Extraction

Data were independently extracted by two reviewers using a standardized data extraction form. Extracted variables included study characteristics, patient demographics, sarcopenia assessment methods, treatment details, and outcomes of interest.

Where available, multivariable‐adjusted effect estimates were extracted preferentially; the univariable estimate was used only when no adjusted estimate was reported, and the adjustment status of every estimate is documented. For each included study we recorded the exact source (table and model) of every extracted hazard ratio, its adjustment status and the covariates included in the adjusted model, the number of outcome events where reported, and the sarcopenia definition, threshold, and threshold source (Table 2). Outcome event counts were reported, or derivable from the reported data, for one study only; this is recorded explicitly in Table 2.

During revision, every entered estimate was independently re‐verified against the source publication by two reviewers. Discrepancies were resolved through consensus.

### 2.7 Outcomes

The primary outcome was overall survival (OS). Secondary outcomes included progression‐free survival (PFS).

### 2.8 Risk of Bias Assessment

Risk of bias was assessed using the Quality in Prognosis Studies (QUIPS) tool [12], which is designed for prognostic factor research and covers six domains: study participation, study attrition, prognostic factor measurement, outcome measurement, study confounding, and statistical analysis and reporting; Two reviewers performed the assessment independently, and disagreements were resolved through discussion. An overall risk‐of‐bias judgment was assigned to each study using the most severe domain‐level judgment, so that a study rated at high risk in any single domain was rated at high risk overall. This rule is deliberately conservative; it was applied uniformly across the three studies. Domain‐level judgments are presented as a traffic‐light figure (Figure 4), and the rationale for each study's domain judgments is reported in the Results; the composite Newcastle–Ottawa Scale scores from the original assessment are retained in Table 1 for transparency.

**TABLE 1:** Summary of baseline characteristics of the three included studies evaluating baseline CT‐defined sarcopenia in HNSCC patients treated with immune checkpoint inhibitors.

Study	*N*	Low muscle mass, *n*	CT level/others	ICI_Type	NOS
Arribas et al. 2021 [8]	61	30	Cross section of L3	AntiPD1 and AntiPDL1	8
Takenaka et al. 2022 [9]	114	31	L3 (SMI)	Nivolumab	7
Kasahara et al. 2026 [11]	179	98	C3‐based (lumbar SMI estimated from cervical CT)	Anti PD‐1 monotherapy and Combined	7

*Note:* For Arribas et al. this is the low‐SMI group defined by a split at the cohort median baseline SMI (30 of 61) and not the sarcopenic group, since that report separately classified 41 of 61 patients (67.2%) as sarcopenic using the Martin et al. cutpoints.

Abbreviations: C3, third cervical vertebra; CT, computed tomography; ICI, immune checkpoint inhibitor; L3, third lumbar vertebra; Low muscle mass, *n*, the number of patients in the exposed group as defined by each individual study; *N*, total sample size; NOS, Newcastle–Ottawa Scale (composite score from the original assessment, retained for reference; domain‐level QUIPS judgments appear in Figure 4); PD‐1/PD‐L1, programmed death (ligand) 1; SMI, skeletal muscle index.

### 2.9 Certainty of Evidence

The certainty of the body of evidence for each outcome was rated using the GRADE approach as adapted for prognostic factor research [10], considering risk of bias, inconsistency, indirectness, imprecision, and publication bias, and is summarized in a summary‐of‐findings table (Table 3).

### 2.10 Statistical Analysis

Hazard ratios (HRs) with corresponding 95% confidence intervals (CIs) were extracted for time‐to‐event outcomes (OS and PFS).

A random‐effects meta‐analysis of log hazard ratios was performed using the Hartung–Knapp–Sidik–Jonkman (HKSJ) method [13]. Because unmodified HKSJ can produce anti‐conservatively narrow intervals when few, highly concordant studies are pooled, the truncated variance correction was applied, taking the larger of the HKSJ standard error and the conventional random‐effects standard error [14]. Between‐study variance (*τ* ^2^) was estimated using restricted maximum likelihood. 95% prediction intervals were computed [15]. Because *τ* ^2^ was estimated at zero for both outcomes, the prediction interval reduces algebraically to the confidence interval evaluated at the same *t* quantile; the two are therefore numerically identical here, and the prediction interval is reported for completeness only. Prediction intervals are in any case not meaningfully estimable when only three studies are available. Leave‐one‐out sensitivity analyses were conducted, as were two further sensitivity analyses: one substituting the univariable for the multivariable overall survival estimate of Arribas et al. [8], and one substituting the cutpoint‐defined (Martin et al.) sarcopenia estimate reported by Arribas et al. for the cohort‐median‐split estimate used in the primary analysis. Subgroup analysis by assessment method was not performed because all included studies used CT‐based assessment and the number of studies was insufficient. Assessment of small‐study effects (funnel plot and regression‐based tests) was not undertaken because fewer than 10 studies were available [7].

All statistical analyses were performed in R (version 4.5.2) using the meta package; pooled estimates were obtained with metagen, and the truncated variance correction was implemented with the adhoc.hakn.ci = “se” option. The analysis script and the complete extraction dataset are openly available.

## 3 Results

Of 441 unique records identified after deduplication, 12 articles underwent full‐text review, and 3 studies were ultimately included in the meta‐analysis (Figure 1), comprising 354 patients (study sample sizes 61–179). During revision, every extracted effect estimate was re‐verified against its source publication, and four studies included in the originally submitted analysis were excluded on re‐verification: one study reported an estimate for baseline cachexia combined with > 2% early weight loss during therapy rather than for CT‐defined sarcopenia [16]; one enrolled only patients who had progressed after ICI therapy and evaluated salvage chemotherapy [17]; one assessed muscle mass by bioelectrical impedance analysis [18] and, additionally, originated from the same department and investigator group as the salvage‐chemotherapy cohort, with the possibility of patient overlap that could not be excluded [17, 18]; and one reported muscle status only as a continuous automated thoracic muscle‐to‐bone ratio without a dichotomous sarcopenia definition [19]. All three included studies were retrospective cohorts of recurrent and/or metastatic HNSCC treated with ICIs using CT‐based skeletal muscle assessment: direct measurement at the third lumbar vertebra (L3) in two studies [8, 9] and lumbar SMI estimated from cervical (C3‐level) CT imaging in one [11]. Study characteristics are summarized in Table 1; the provenance, adjustment status, and sarcopenia thresholds of every extracted estimate are given in Table 2. The proportion of patients in the exposed (low muscle mass) group ranged from 27% (31/114) in Takenaka et al. [9] through 49% (30/61) in Arribas et al. [8] to 55% (98/179) in Kasahara et al. [11]. This range reflects the different thresholds applied, a split at the cohort median in Arribas et al., sex‐specific cutoffs derived within the cohort by time‐dependent ROC analysis in Takenaka et al., and a fixed external cutoff of 43.2 cm^2^/m^2^ in Kasahara et al., rather than differences in the underlying populations alone.

**TABLE 2:** Provenance, adjustment status, adjusted covariates, outcome events, and sarcopenia definitions and thresholds of all extracted effect estimates. Where the source report did not state the number of outcome events and it could not be derived from the reported data, this is recorded as not reported.

Study	Outcome	HR (95% CI) as entered	Source in original report, model and adjustment covariates	Outcome events (*n*)	Sarcopenia definition, threshold and threshold source
Arribas et al. 2021 [8]	OS	2.19 (1.19–4.05)	Table 2 (multivariate analysis), overall survival column. Multivariable Cox, adjusted for age, serum albumin and platinum‐refractory status.	45 deaths (16 of 61 alive at last follow‐up)	L3 SMI on axial CT (SliceOmatic; −29 to +150 HU); dichotomized at the cohort median baseline SMI of 42.0 cm^2^/m^2^ (IQR 37.5–48.6). Threshold internally derived; not externally validated.
Arribas et al. 2021 [8]	PFS	1.84 (1.08–3.12)	Table 2 (univariate analysis), global PFS column. Univariable; no multivariable global PFS model was computed by the source authors because of the small number of patients in the no‐event group.	Not reported	As above
Takenaka et al. 2022 [9]	OS	2.06 (1.16–3.67)	Table 2 (Cox proportional hazards analysis for overall survival), multivariate column. Multivariable Cox, adjusted for subcutaneous adipose index, visceral adipose index, body mass index and ECOG performance status.	Not reported	L3 SMI on abdominal CT (BMI_CT_ver6 software); sex‐specific thresholds of 39.84 cm^2^/m^2^ for men and 35.92 cm^2^/m^2^ for women, derived within the same cohort by time‐dependent ROC analysis.
Takenaka et al. 2022 [9]	PFS	1.74 (1.04–2.92)	Table 3 (Cox proportional hazards analysis for progression‐free survival), multivariate column. Multivariable Cox, adjusted for subcutaneous adipose index, visceral adipose index, body mass index and primary tumor site.	Not reported	As above
Kasahara et al. 2026 [11]	OS	1.94 (1.15–3.27)	Table 2, multivariate column for overall survival. Multivariable Cox, adjusted for body mass index, neutrophil‐to‐lymphocyte ratio and platelet‐to‐lymphocyte ratio.	Not reported	Lumbar SMI estimated from a C3‐level cervical CT slice using the Swartz regression equation, which incorporates age, weight and sex; sarcopenia defined as LSMI < 43.2 cm^2^/m^2^. Threshold taken from previously published head and neck cancer cohorts (Wendrich et al. 2017; Yamahara et al. 2021) as cited in the source report.
Kasahara et al. 2026 [11]	PFS	1.92 (1.33–2.75)	Table 2, multivariate column for progression‐free survival. Multivariable Cox, adjusted for age, primary tumor site and neutrophil‐to‐lymphocyte ratio.	Not reported	As above

The included cohorts differed in setting and case mix. Arribas et al. [8] enrolled 61 consecutive patients at a single Spanish center between July 2015 and December 2018 (mean age 57.7 years, 85.2% male; 63.9% received an ICI as second or later line; 59.0% were platinum‐refractory; median follow‐up 9 months). Takenaka et al. [9] enrolled 114 patients across three Japanese centers between March 2017 and June 2020 (median age 65 years, 74.6% male; 15.8% HPV‐positive; nivolumab was first‐line therapy for recurrent or metastatic disease in 43.9%; median follow‐up 23.1 months among surviving patients). Kasahara et al. [11] enrolled 179 patients at Japanese centers between April 2017 and July 2024 (median age 70 years, 86.6% male; 79.8% had received prior chemotherapy; median overall survival 20.4 months). All three were retrospective cohorts of recurrent and/or metastatic disease. ECOG performance status was recorded by Arribas et al. and Takenaka et al. but entered the multivariable model only in Takenaka et al., and was not consistently available in Kasahara et al. HPV or p16 status was reported systematically only by Takenaka et al.; Arribas et al. noted HPV‐related disease in three oropharyngeal cases, and HPV status was unavailable in Kasahara et al. PD‐L1 combined positive score was not reported in any of the three cohorts. Neither Takenaka et al. nor Kasahara et al. reported the number of deaths or progression events, and these could not be derived from the published data.

Note on the Arribas et al. [8] overall survival estimate. The abstract of that report gives a multivariate overall survival HR of 2.99 (95% CI 1.29–6.94), whereas its Results text and its Table 2 give 2.19 (95% CI 1.19–4.05) for the same model, adjusted for age, serum albumin, and platinum‐refractory status. The value from the body of the report (2.19) was entered, in accordance with our pre‐specified rule of extracting from the full text in preference to the abstract. The discrepancy is recorded here so that readers and any subsequent meta‐analyst working from abstracts can reconcile the two figures.

**FIGURE 2:**
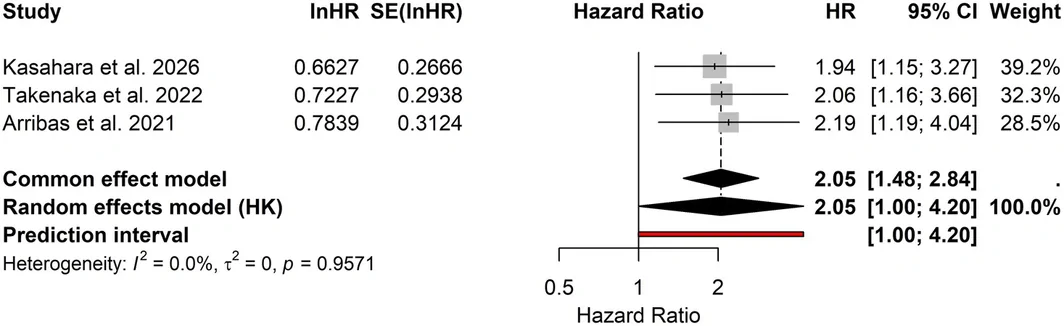
Forest plot showing the pooled hazard ratio (HR) for overall survival (OS) comparing patients with sarcopenia versus non‐sarcopenia in HNSCC treated with immune checkpoint inhibitors. Hazard ratios and confidence limits are plotted from the log hazard ratio and its standard error. The recomputed limits may therefore differ in the second decimal place from the limits printed in the source reports and recorded in Table 2, the upper limit for Arribas et al. [8] is 4.04 here and 4.05 in the source, because the published intervals are marginally asymmetric on the log scale once the point estimate is rounded. Table 2 records the source values as printed.

For overall survival, three studies were pooled. Sarcopenia was associated with worse OS (HKSJ random‐effects HR 2.05, 95% CI 1.00–4.20; common‐effect HR 2.05, 95% CI 1.48–2.84; *I* ^2^ = 0%, *τ* ^2^ = 0, *Q*‐test *p* = 0.957) (Figure 2). The width of the HKSJ interval reflects the use of a *t* distribution with two degrees of freedom when only three studies are pooled; the point estimate was stable across models. The lower limit of the HKSJ interval was 0.9985, so the primary overall survival analysis did not reach conventional statistical significance. The 95% prediction interval (1.00–4.20) is numerically identical to the HKSJ confidence interval. This is an algebraic consequence of *τ* ^2^ being estimated at zero rather than a transcription error: with no between‐study variance the prediction interval reduces to the confidence interval on the same *t* quantile. It therefore conveys no information beyond the confidence interval and is not meaningfully estimable from three studies. A sensitivity analysis using the univariable rather than the multivariable estimate from Arribas et al. [8] yielded a pooled HR of 2.01 (95% CI 0.99–4.10). A second sensitivity analysis addressed the difference in exposure definition between the included cohorts. Arribas et al. [8] dichotomized at the cohort median baseline SMI, but also report a multivariable overall survival HR of 2.06 (95% CI 1.01–4.23) for sarcopenia defined by the Martin et al. cutpoints and adjusted for the same covariates; substituting that estimate yielded a pooled HR of 2.01 (95% CI 0.95–4.24; common‐effect HR 2.01, 95% CI 1.43–2.82; *I* ^2^ = 0%), leaving the point estimate essentially unchanged.

**FIGURE 3:**
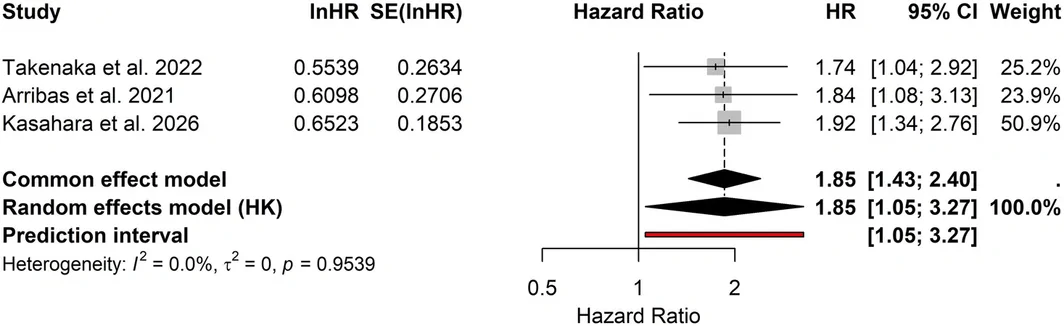
Forest plot depicting the pooled hazard ratio (HR) for progression‐free survival (PFS) in patients with and without sarcopenia undergoing immunotherapy. Confidence limits are recomputed from the log hazard ratio and its standard error, as described for Figure 2.

For progression‐free survival, the same three studies contributed estimates. Sarcopenia was associated with worse PFS (HKSJ random‐effects HR 1.85, 95% CI 1.05–3.27; common‐effect HR 1.85, 95% CI 1.43–2.40; *I* ^2^ = 0%) (Figure 3). Given the small number of studies and the dominant weight of a single study (Kasahara et al., 50.9%), the PFS finding should be regarded as hypothesis‐generating. The 95% prediction interval was 1.05–3.27, again numerically identical to the confidence interval for the reason given above.

Assessment of small‐study effects was not performed, as the number of included studies (three) is well below the conventional minimum of approximately 10 required for funnel‐plot‐based methods [7].

### 3.1 Risk of Bias

**FIGURE 4:**
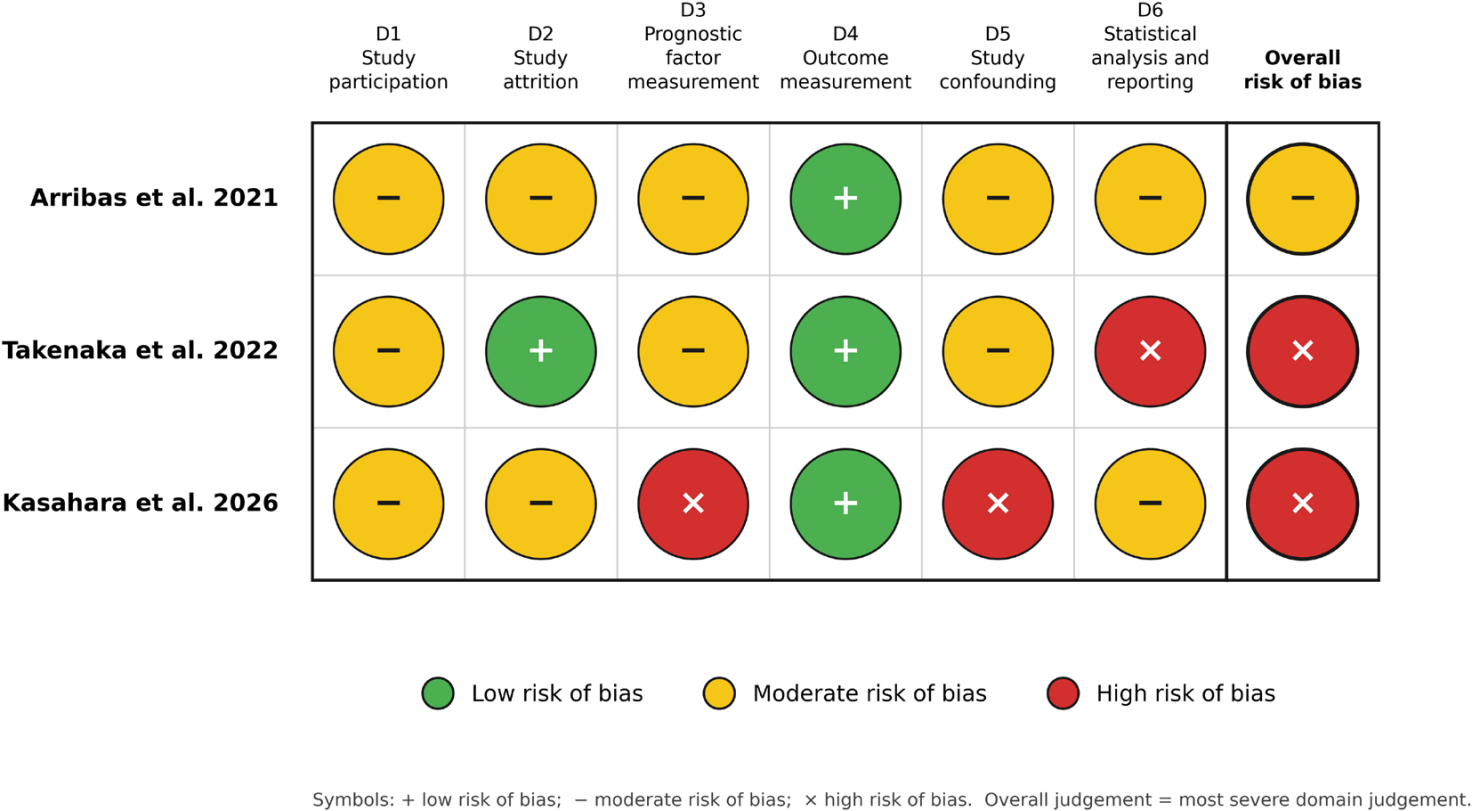
Risk of bias assessment.

Applying the most‐severe‐domain rule described in the Methods, the overall QUIPS judgment was moderate risk of bias for Arribas et al. [8] and high risk of bias for both Takenaka et al. [9] and Kasahara et al. [11] (Figure 4). Outcome measurement was the only domain judged at low risk in all three studies, each of which ascertained death and progression by radiological assessment, from complete institutional records.

For Arribas et al. [8] no domain was judged at high risk. Study participation, study attrition, prognostic factor measurement, study confounding, and statistical analysis and reporting were each judged at moderate risk: the cohort was consecutively accrued at a single center but small (*n* = 61); image analysis was performed by a single observer blinded to patient data, but the exposure was defined by a split at the cohort median rather than by an externally derived threshold; and the adjusted overall survival model included only age, serum albumin, and platinum‐refractory status, leaving performance status, HPV/p16 status, and tumor burden unaccounted for.

For Takenaka et al. [9] the high overall judgment was driven by a single domain, statistical analysis and reporting: the sex‐specific SMI thresholds were derived by time‐dependent ROC analysis in the same dataset subsequently used for the survival models, a risk of overfitting that the authors themselves acknowledge. Study attrition was judged at low risk. Study participation was judged at moderate risk because 41 of 157 otherwise eligible patients were excluded for want of an abdominal CT image at L3.

For Kasahara et al. [11] the high overall judgment was driven by two domains, prognostic factor measurement and study confounding. Lumbar SMI was not measured directly but estimated from a C3‐level cervical slice using a published regression equation that incorporates age, weight, and sex, so that the exposure is in part a function of covariates; and performance status, comorbidity, smoking, alcohol consumption, HPV status, and PD‐L1 combined positive score were, by that study's own account, not consistently available and were not adjusted for.

The study judged at high risk of bias for prognostic factor measurement and confounding carries 39.2% of the weight for overall survival and 50.9% of the weight for progression‐free survival. This is taken into account in the certainty rating below.

**FIGURE 5:**
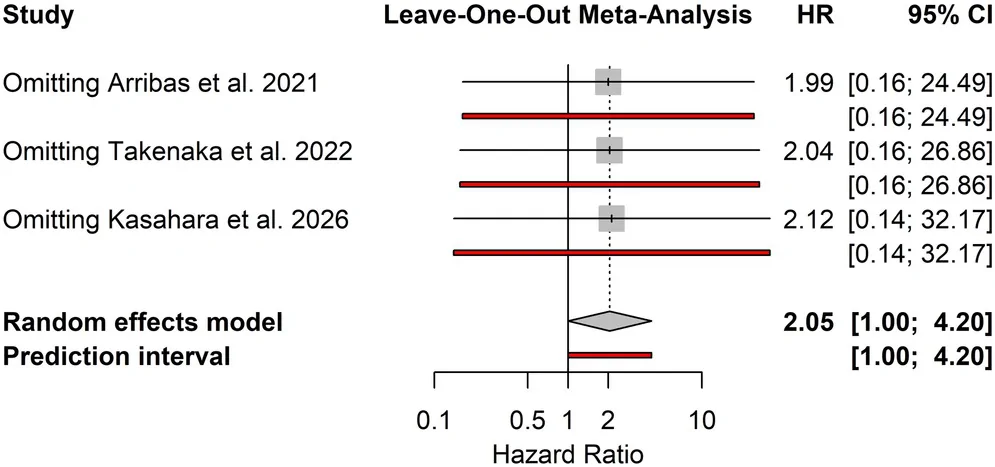
Leave‐one‐out sensitivity analysis for overall survival (descriptive; two studies per iteration).

Leave‐one‐out analysis was consistent in the direction of effect (pooled OS HRs 1.99–2.12 across iterations); however, with only two studies remaining in each iteration, HKSJ confidence intervals were extremely wide (*t* distribution with one degree of freedom) and these analyses are reported descriptively (Figure 5).

The direction of effect for PFS was likewise consistent across leave‐one‐out iterations, with the same caveat regarding interval width at *k* = 2.

### 3.2 Certainty of Evidence

Using GRADE as adapted for prognostic factor reviews [10], the certainty of evidence was rated low for OS and low for PFS. Both outcomes were downgraded one level for risk of bias and one level for imprecision (three studies, with HKSJ intervals crossing or approaching the null). The risk‐of‐bias downgrade was limited to one level notwithstanding the high overall judgment for two of the three studies, for four reasons. Under the most‐severe‐domain rule adopted here a single domain determines the overall judgment, and in each case only one or two of six domains were rated high. No study was at high risk for outcome measurement or attrition, so the time‐to‐event data themselves are unlikely to be biased. The mechanisms driving the two high judgments—a threshold derived within the analysis dataset in Takenaka et al. [9], and an estimated rather than directly measured exposure with residual confounding in Kasahara et al. [11]—bear principally on the precision and transportability of the thresholds rather than on the direction of the association. And the three point estimates were closely concordant (*I* ^2^ = 0%, *Q*‐test *p* = 0.957), which is not the pattern expected if study‐specific bias were driving the result. We note that a two‐level downgrade for risk of bias would yield very low certainty for both outcomes, that this judgment is finely balanced given the weight carried by the study at high risk for confounding, and that the clinical inference is unchanged either way. No downgrade was applied for inconsistency (*I* ^2^ = 0%, concordant point estimates) or indirectness (Table 3).

**TABLE 3:** GRADE summary of findings for the certainty of evidence, adapted for prognostic factor reviews [10].

Outcome	Studies (patients)	Pooled HR (95% CI), HKSJ truncated	Certainty (GRADE)	Rationale
Overall survival	3 (354)	2.05 (1.00–4.20)	Low ⊕⊕◯◯	Downgraded one level for risk of bias (all three studies retrospective; two at high overall risk on QUIPS—Takenaka et al., threshold derived within the analysis dataset; Kasahara et al., estimated exposure with performance status, HPV status and PD‐L1 unadjusted—with variable, incompletely reported adjustment) and one level for imprecision (*k* = 3; interval crosses null under HKSJ). The justification for a one‐ rather than two‐level risk‐of‐bias downgrade is given in the Results. Not downgraded for inconsistency (*I* ^2^ = 0%) or indirectness.
Progression‐free survival	3 (354)	1.85 (1.05–3.27)	Low ⊕⊕◯◯	Downgraded one level for risk of bias (as for overall survival; the study carrying 50.9% of the weight is at high overall risk) and one level for imprecision (*k* = 3; the interval excludes the null only narrowly); hypothesis‐generating.

## 4 Discussion

Our corrected meta‐analysis of three retrospective cohorts indicates that baseline CT‐defined sarcopenia is associated with poorer overall survival (pooled HR 2.05) and progression‐free survival (pooled HR 1.85) in patients with HNSCC treated with ICIs, although with only three studies the HKSJ confidence interval for OS marginally crosses unity. The direction and magnitude of effect were highly concordant across the included studies. Takenaka et al. [9] reported that low skeletal muscle index predicted worse OS and PFS in nivolumab‐treated HNSCC, Arribas et al. [8] found low SMI to be an independent factor for OS on multivariable analysis, and Kasahara et al. [11] confirmed that sarcopenia independently predicted both OS and PFS, whereas systemic inflammatory markers lost significance in adjusted analyses, suggesting that skeletal muscle depletion may provide stronger prognostic discrimination than inflammation‐based indices alone.

Related evidence that fell outside our eligibility criteria points in the same direction. Jungbauer et al. [19], excluded from pooling because muscle status was analyzed only as a continuous automated thoracic muscle‐to‐bone ratio, reported that a higher ratio was independently associated with longer overall survival and that interval decline in this parameter predicted markedly poorer survival. Willemsen et al. [16], excluded because the reported estimate combined baseline cachexia with early weight loss during therapy, similarly suggested that ongoing catabolic activity carries prognostic information beyond baseline body composition. These observations support the broader construct of depleted muscle reserve as an adverse host phenotype in ICI‐treated HNSCC, while underscoring why standardized, baseline, CT‐based definitions are needed before such measures can be pooled.

The biological plausibility of this association is reinforced by the broader HNSCC literature linking skeletal muscle depletion with frailty, cachexia, and impaired physiologic reserve. Zwart et al. [6] found that CT‐defined sarcopenia was independently associated with frailty, while Chargi et al. [20] showed that sarcopenia predicted survival in elderly head and neck cancer patients. Collectively, these findings support the interpretation that sarcopenia in HNSCC is not merely an anatomic measurement but a clinically meaningful marker of systemic vulnerability that may influence tolerance of therapy and capacity for durable anti‐tumor benefit.

While this manuscript was under review, an independent systematic review and meta‐analysis of the same question was published. Kono et al. [21] pooled four studies restricted to CT‐based, multivariable‐adjusted estimates and reported a pooled OS HR of 2.05 (95% CI 1.48–2.84) with no heterogeneity. Our corrected common‐effect estimate is 2.05 (95% CI 1.48–2.84), identical to theirs to three significant figures; recomputed from the three entered estimates it is 2.0476, with limits 1.4763 and 2.8397. We state plainly that this exact agreement is a coincidence. The two analyses rest on overlapping but non‐identical evidence bases, and an identity to three digits between a three‐study and a four‐study synthesis carries no more evidential weight than close concordance would. The two reviews were conducted independently by unrelated author groups, and our search window closed before Kono et al. appeared. Reconciling the two included‐study lists, three studies are common to both syntheses (Arribas et al. [8], Takenaka et al. [9] and Kasahara et al. [11]); the fourth study pooled by Kono et al.

Willemsen et al. [16], which was retrieved by our search, assessed in full text, and excluded under exposure‐related exclusion criterion (iii) because its reported estimate combined baseline cachexia with > 2% early weight loss during therapy rather than baseline CT‐defined sarcopenia. The incremental contribution of the present work is therefore an independent verification of the OS association based on an explicitly re‐verified extraction with documented estimate provenance, together with a quantitative synthesis of PFS, which Kono et al. did not analyze. We acknowledge that, with three studies, this increment is modest, and we have calibrated our conclusions accordingly.

An important interpretive caveat is that all included studies were single‐arm cohorts of ICI‐treated patients without a non‐ICI comparator. Sarcopenia is an adverse prognostic factor in HNSCC treated with surgery, chemoradiation, and chemotherapy alike [1, 20]; the present data therefore establish a prognostic association within ICI‐treated populations and cannot test a treatment‐by‐sarcopenia interaction. These findings must not be interpreted as evidence for withholding immunotherapy from patients with sarcopenia.

From a practical perspective, our results support evaluating muscle status from routinely available imaging as part of baseline prognostic assessment in ICI‐treated HNSCC. This is particularly relevant in head and neck oncology, where standard abdominal imaging is often unavailable and alternative measures such as cervical (C3‐based), craniofacial, or automated thoracic body composition analysis may be more feasible; indeed, one included study derived lumbar SMI from cervical CT [11]. Balanta‐Melo et al. [22] showed that masseter thickness may serve as an alternative biomarker of cachexia, further supporting the concept that clinically useful body composition assessment need not be limited to a single anatomical landmark. Nevertheless, cross‐level measurement harmonization (L3, C3, thoracic surrogates) remains an open methodological problem, and the current evidence base is limited by retrospective designs and heterogeneous thresholds, underscoring the need for prospective studies using standardized imaging definitions.

While multiple systematic reviews and meta‐analyses have evaluated the prognostic significance of sarcopenia across various malignancies, including gastric [23], pancreatic [24], and ovarian cancers [25], evidence in ICI‐treated HNSCC remains limited: our synthesis and the independent review by Kono et al. [21] converge on an approximately two‐fold increase in the hazard of death associated with baseline CT‐defined sarcopenia. This review has several limitations. The evidence base comprises only three retrospective cohorts; adjustment for prognostic covariates was variable and incompletely reported; sarcopenia thresholds were study‐specific; and the original search was restricted to three databases, which has been maintained. Reflecting these limitations, we rate the certainty of evidence as low for both outcomes, and the findings should be interpreted with corresponding caution. Large‐scale prospective studies with standardized assessment methods, and ideally individual‐participant‐data collaboration, are required to establish definitive clinical utility.

The search itself is a further and material limitation. Only the Scopus strategy incorporated an immune checkpoint inhibitor concept; the PubMed and Cochrane Library strategies were built on population and body‐composition terms alone, and the intervention restriction was applied by manual screening. No controlled vocabulary was used in any database, and Embase and Web of Science were not searched. Eligible reports indexed only in those databases, or retrievable only through controlled vocabulary or an intervention‐specific term, may therefore have been missed, and we cannot exclude that the evidence base assembled here is incomplete. The search could not be re‐run within the timeframe of this revision, so the limitation is reported rather than remedied; the reconciliation against the included‐study list of Kono et al. [21] above should be read as a partial check on completeness rather than as a substitute for a fully specified search.

A second inconsistency should be stated plainly. The overall survival estimate entered for Arribas et al. [8] derives from a split at that cohort's own median baseline SMI, whereas Takenaka et al. [9] and Kasahara et al. [11] used pre‐specified thresholds. Pooling these requires only the assumption that each study contrasts a lower‐muscle‐mass with a higher‐muscle‐mass stratum of its own cohort, which is the contrast of interest here; but it does mean that the pooled estimate does not correspond to a single, transportable definition of sarcopenia, and that the exposure prevalences in Table 1 are not comparable across studies. The sensitivity analysis substituting the cutpoint‐defined Arribas estimate (Results) shows that the pooled result is not driven by this choice.

## 5 Conclusion

In three retrospective cohorts totaling 354 patients, baseline CT‐defined sarcopenia was associated with an approximately two‐fold increase in the hazard of death, and of progression or death, in patients with HNSCC receiving immune checkpoint inhibitors. This association should be stated with the precision the data support. Under the pre‐specified primary estimator the 95% confidence interval for overall survival marginally included the null (HR 2.05, 95% CI 1.00–4.20), so the primary overall survival analysis did not reach conventional statistical significance, and the progression‐free survival interval excluded the null only narrowly (HR 1.85, 95% CI 1.05–3.27). The association rests on the size and consistency of the point estimates, the complete absence of heterogeneity, the common‐effect interval (1.48–2.84 for overall survival) and concordance with an independent published synthesis, rather than on the primary interval alone. Certainty of evidence is low for both outcomes. Sarcopenia should therefore currently be regarded as a prognostic marker for risk stratification rather than a predictive biomarker, and must not be used to justify withholding immunotherapy. Prospective validation with standardized CT‐based definitions is required.

## Author Contributions

**Diwakar Koirala:** conceptualization, writing – original draft, writing – review and editing, funding acquisition, visualization. **Nakendra Malla:** conceptualization. **Bivek Mishra:** methodology. **Ramesh Sapkota:** software, data curation. **Sahil Niraula:** investigation, validation. **Kushal Bastola:** formal analysis, funding acquisition. **Nirman Khatiwada:** investigation, writing – review and editing. **Manish Gahatraj:** writing – review and editing, writing – original draft. **Rijan Kafle:** writing – review and editing.

## Funding

The authors have nothing to report.

## Ethics Statement

The authors have nothing to report.

## Consent

The authors have nothing to report.

## Conflicts of Interest

The authors declare no conflicts of interest.

## Supporting information


**Data S1:** Supporting Information 1.

**Data S2:** Supporting Information 2.

**Data S3:** PRISMA checklist.

## Data Availability

The complete data extraction sheet, the per‐study estimate provenance table, the risk‐of‐bias judgments, and the R analysis script are openly available in a public repository at: https://doi.org/10.17605/OSF.IO/HUZJE.
