# Supplementary material for: Sarcopenia and Survival in Patients With Head and Neck Squamous Cell Carcinoma Receiving Immune Checkpoint Inhibitors: A Systematic Review and Meta‐Analysis of Prognostic Association
Source: Cancer Rep (Hoboken). 2026 Sep 21;9(9):e70695. doi: 10.1002/cnr2.70695 (PMC13593368; doi:10.1002/cnr2.70695)
Supplement: Supplementary file 1 [file CNR2-9-e70695-s001.pdf]

Pubmed

("head and neck squamous cell carcinoma" OR HNSCC OR "head and neck cancer") AND (sarcopenia OR "skeletal muscle index" OR "skeletal muscle area" OR "muscle mass")

Cochrane:

("head and neck squamous cell carcinoma" OR HNSCC OR "head and neck cancer" OR "oral cancer" OR "oropharyngeal cancer" OR "laryngeal cancer" OR "hypopharyngeal cancer")

AND

(sarcopenia OR "skeletal muscle index" OR SMI OR "skeletal muscle area" OR "muscle mass" OR "muscle depletion" OR "body composition")

"head and neck cancer" "skeletal muscle index"

Scopus:

TITLE-ABS-KEY(

"head and neck squamous cell carcinoma"

OR "head and neck cancer"

OR "oral cancer"

OR "oropharyngeal cancer"

OR "laryngeal cancer"

OR "hypopharyngeal cancer"

OR hnscc

)

AND

TITLE-ABS-KEY(

sarcopenia

OR "muscle mass"

OR "body composition"

OR "skeletal muscle"

OR "skeletal muscle index"

OR "skeletal muscle area"

OR "muscle depletion"

)

```
AND
TITLE-ABS-KEY(
  "immune checkpoint inhibitor*"
  OR immunotherapy
  OR "PD-1"
  OR "PD-L1"
  OR "programmed death 1"
  OR "programmed death ligand 1"
  OR nivolumab
  OR pembrolizumab
  OR cemiplimab
  OR atezolizumab
  OR durvalumab
  OR ipilimumab
  OR "CTLA-4"
)
```
