# Supplementary material for: Sarcopenia and Survival in Patients With Head and Neck Squamous Cell Carcinoma Receiving Immune Checkpoint Inhibitors: A Systematic Review and Meta‐Analysis of Prognostic Association
Source: Cancer Rep (Hoboken). 2026 Sep 21;9(9):e70695. doi: 10.1002/cnr2.70695 (PMC13593368; doi:10.1002/cnr2.70695)
Supplement: Supplementary file 2 [file CNR2-9-e70695-s002.docx]

**Supplementary Material S2**

**Manuscript ID:** 9646802

**Manuscript title:** Sarcopenia and survival in patients with head and neck squamous cell carcinoma receiving immune checkpoint inhibitors: a systematic review and meta-analysis of prognostic association

**Corresponding author:** Dr. Diwakar Koirala, B.P. Koirala Institute of Health Sciences, Dharan, Nepal

**Scope**

Of 441 unique records screened after deduplication, 12 reports were sought for retrieval and assessed for eligibility in full text. Three met all eligibility criteria and were included in the quantitative synthesis; nine were excluded. Every excluded report is cited individually below with its primary reason for exclusion and the specific eligibility criterion it failed. The counts reconcile with the PRISMA flow diagram (Figure 1) as follows: 5 + 1 + 1 + 1 + 1 = 9.

Reports E6 to E9 were included in the originally submitted version of this manuscript and were excluded during revision, after every extracted effect estimate was independently re-verified against its source publication by two reviewers. This is declared as a protocol deviation in the main text (Methods, Protocol Deviations).

**Excluded reports (n = 9)**

| **ID** | **Report assessed in full text** | **Primary reason for exclusion** | **Eligibility criterion not met** |
| --- | --- | --- | --- |
| **E1** | Chargi N, Bril SI, Emmelot-Vonk MH, de Bree R. Sarcopenia is a prognostic factor for overall survival in elderly patients with head-and-neck cancer. Eur Arch Otorhinolaryngol. 2019;276(5):1475–86. doi:10.1007/s00405-019-05361-4. PMID: 30830300; PMCID: PMC6458984. | No immune checkpoint inhibitor–treated subgroup and no ICI-specific prognostic estimate extractable for the sarcopenic versus non-sarcopenic comparison. The cohort comprises elderly patients with head and neck cancer treated with curative-intent modalities. | Population: no ICI-treated cohort |
| **E2** | Lee B, Bae YJ, Jeong WJ, Kim H, Choi BS, Kim JH. Temporalis muscle thickness as an indicator of sarcopenia predicts progression-free survival in head and neck squamous cell carcinoma. Sci Rep. 2021;11(1):19717. doi:10.1038/s41598-021-99201-3. PMCID: PMC8492642. | No immune checkpoint inhibitor–treated subgroup and no ICI-specific prognostic estimate extractable for the sarcopenic versus non-sarcopenic comparison. The cohort comprises patients with newly diagnosed HNSCC. Muscle status was additionally assessed as temporalis muscle thickness rather than a dichotomised skeletal muscle index or area. | Population: no ICI-treated cohort |
| **E3** | Balanta-Melo J, Jones AJ, Novinger LJ, Moore MG, Bonetto A. Masseter muscle thickness is predictive of cancer cachexia in patients with head and neck cancer. Head Neck. 2024;46(11):2678–86. doi:10.1002/hed.27789. PMID: 38661244; PMCID: PMC11473234. | No immune checkpoint inhibitor–treated subgroup and no ICI-specific prognostic estimate extractable for the sarcopenic versus non-sarcopenic comparison. Muscle status was additionally assessed as masseter muscle thickness rather than a dichotomised skeletal muscle index or area, and the reported outcome was cancer cachexia rather than survival. | Population: no ICI-treated cohort |
| **E4** | Huiskamp LFJ, Chargi N, Devriese LA, de Jong PA, de Bree R. The predictive and prognostic value of low skeletal muscle mass for dose-limiting toxicity and survival in head and neck cancer patients receiving concomitant cetuximab and radiotherapy. Eur Arch Otorhinolaryngol. 2020;277(10):2847–58. doi:10.1007/s00405-020-05972-2. PMID: 32335709; PMCID: PMC7496017. | No immune checkpoint inhibitor–treated subgroup and no ICI-specific prognostic estimate extractable for the sarcopenic versus non-sarcopenic comparison. The cohort received concomitant cetuximab and radiotherapy; cetuximab is an anti-EGFR monoclonal antibody and not an immune checkpoint inhibitor. Muscle mass was measured at the third cervical vertebra on CT or MRI, with the threshold derived from the lowest log-likelihood for cetuximab dose-limiting toxicity. | Population: no ICI-treated cohort |
| **E5** | Kasahara K, Shigetomi S, Sato Y, Imanishi Y, Ikari Y, Nishiyama T, et al. Sarcopenia as a predictive factor for febrile neutropenia during induction chemotherapy in head and neck squamous cell cancer. Auris Nasus Larynx. 2024;51(6):971–5. doi:10.1016/j.anl.2024.09.010. | No immune checkpoint inhibitor–treated subgroup and no ICI-specific prognostic estimate extractable for the sarcopenic versus non-sarcopenic comparison. The cohort (n = 71) received induction chemotherapy, not immune checkpoint inhibitors, and the reported outcome was febrile neutropenia rather than overall or progression-free survival. Muscle mass was measured on cervical CT with lumbar SMI estimated by the Swartz equation and a threshold of 43.2 cm²/m². | Population: no ICI-treated cohort; outcome not OS or PFS |
| **E6** | Willemsen ACH, De Moor N, Van Dessel J, Baijens LWJ, Bila M, Hauben E, et al. The predictive and prognostic value of weight loss and body composition prior to and during immune checkpoint inhibition in recurrent or metastatic head and neck cancer patients. Cancer Med. 2023;12(7):7699–712. doi:10.1002/cam4.5522. PMID: 36484469. | Exposure was not baseline CT-defined sarcopenia. The reported hazard ratio corresponds to baseline cachexia combined with >2% early weight loss during therapy, independent of PD-L1 expression; the exposure therefore incorporates on-treatment change and is not a dichotomised CT-based SMI or SMA. Discussed narratively in the main text. | Exposure criterion (iii): exposure definitions incorporating on-treatment change |
| **E7** | Abe D, Kimura T, Koike S, Tamaki H, Konya S, Kawamoto M, et al. The role of salvage chemotherapy on survival outcomes following immune checkpoint inhibitor therapy in recurrent and/or metastatic head and neck squamous cell carcinoma. In Vivo. 2025;39(5):2993–3000. doi:10.21873/invivo.14100. PMID: 40877167; PMCID: PMC12396031. | Analysis population restricted to patients who had already progressed after immune checkpoint inhibitor therapy, with salvage chemotherapy as the exposure of interest. This is not the review population. See also the cohort-overlap note below. | Exposure criterion (iv): cohorts restricted to progression after ICI therapy |
| **E8** | Kimura T, Abe D, Kitano M, Koike S, Umemoto M, Miyamoto K, et al. Prognostic impact of sarcopenia on clinical outcomes in patients with recurrent and/or metastatic head and neck squamous cell carcinoma treated with immune checkpoint inhibitors. Cancer Diagn Progn. 2025;5(5):597–605. doi:10.21873/cdp.10474. PMID: 40900882; PMCID: PMC12401036. | Muscle mass assessed by bioelectrical impedance analysis rather than computed tomography. Independently of this, patient overlap with report E7 could not be excluded. See also the cohort-overlap note below. | Exposure criterion (i): non-radiological assessment of muscle mass |
| **E9** | Jungbauer F, Ludwig S, Huber L, Affolter A, Lammert A, Rotter N, et al. Muscle matters: automated CT-based body composition analysis predicts survival in patients with head and neck cancer treated with immunotherapy. Front Oncol. 2026;16:1725892. doi:10.3389/fonc.2026.1725892. | Muscle status reported only as a continuous automated thoracic muscle-to-bone ratio, with no dichotomous sarcopenia definition and no reported number of sarcopenic patients; no estimate is available for the sarcopenic versus non-sarcopenic contrast. Discussed narratively in the main text. | Exposure criterion (ii): muscle status reported only as a continuous index |

**Note on cohort overlap**

Reports E7 (Abe et al. 2025) and E8 (Kimura et al. 2025) originate from the same Department of Otolaryngology–Head and Neck Surgery (Kindai University), share eight authors, and describe contemporaneous retrospective series of recurrent and/or metastatic HNSCC treated with immune checkpoint inhibitors. Patient overlap between the two cohorts therefore cannot be excluded. Each report was independently ineligible on exposure or population grounds, as stated above, so no decision on which cohort to retain was required. All reports assessed in full text were screened for cohort overlap by cross-checking author lists, institutional affiliations, and accrual periods (Methods, Study Selection). Report E5 (Kasahara et al. 2024) shares its first author and institution with an included study (Kasahara et al. 2026); the two cohorts were compared and found to be distinct, the excluded report comprising patients treated with induction chemotherapy and the included study patients treated with immune checkpoint inhibitors.

**Abbreviations**

CT, computed tomography; HNSCC, head and neck squamous cell carcinoma; ICI, immune checkpoint inhibitor; PD-L1, programmed death-ligand 1; PRISMA, Preferred Reporting Items for Systematic Reviews and Meta-Analyses; SMA, skeletal muscle area; SMI, skeletal muscle index.
