# Supplementary material for: Sarcopenia and Survival in Patients With Head and Neck Squamous Cell Carcinoma Receiving Immune Checkpoint Inhibitors: A Systematic Review and Meta‐Analysis of Prognostic Association
Source: Cancer Rep (Hoboken). 2026 Sep 21;9(9):e70695. doi: 10.1002/cnr2.70695 (PMC13593368; doi:10.1002/cnr2.70695)
Supplement: Supplementary file 3 [file CNR2-9-e70695-s003.docx]

| **Section and Topic** | **Item #** | **Checklist item** | **Location where item is reported** |
| --- | --- | --- | --- |
| **TITLE** | | |  |
| Title | 1 | Identify the report as a systematic review. | Title page, p. 1 |
| **ABSTRACT** | | |  |
| Abstract | 2 | See the PRISMA 2020 for Abstracts checklist. | Abstract, p. 1–2 |
| **INTRODUCTION** | | |  |
| Rationale | 3 | Describe the rationale for the review in the context of existing knowledge. | Introduction, p. 2–3 |
| Objectives | 4 | Provide an explicit statement of the objective(s) or question(s) the review addresses. | Introduction, final paragraph, p. 3 |
| **METHODS** | | |  |
| Eligibility criteria | 5 | Specify the inclusion and exclusion criteria for the review and how studies were grouped for the syntheses. | Methods, Eligibility Criteria, p. 3–4 |
| Information sources | 6 | Specify all databases, registers, websites, organisations, reference lists and other sources searched or consulted to identify studies. Specify the date when each source was last searched or consulted. | Methods, Search Strategy, p. 4 (databases, platforms, last-search dates, reference-list screening); Supplementary material S1 |
| Search strategy | 7 | Present the full search strategies for all databases, registers and websites, including any filters and limits used. | Methods, Search Strategy, p. 4; full search strings as run, per database, in Supplementary material S1 |
| Selection process | 8 | Specify the methods used to decide whether a study met the inclusion criteria of the review, including how many reviewers screened each record and each report retrieved, whether they worked independently, and if applicable, details of automation tools used in the process. | Methods, Study Selection, p. 4 |
| Data collection process | 9 | Specify the methods used to collect data from reports, including how many reviewers collected data from each report, whether they worked independently, any processes for obtaining or confirming data from study investigators, and if applicable, details of automation tools used in the process. | Methods, Data Extraction, p. 5 |
| Data items | 10a | List and define all outcomes for which data were sought. Specify whether all results that were compatible with each outcome domain in each study were sought (e.g. for all measures, time points, analyses), and if not, the methods used to decide which results to collect. | Methods, Outcomes, p. 5–6 |
|  | 10b | List and define all other variables for which data were sought (e.g. participant and intervention characteristics, funding sources). Describe any assumptions made about any missing or unclear information. | Methods, Data Extraction, p. 5; Table 1, p. 7; Table 2, p. 8–10 |
| Study risk of bias assessment | 11 | Specify the methods used to assess risk of bias in the included studies, including details of the tool(s) used, how many reviewers assessed each study and whether they worked independently, and if applicable, details of automation tools used in the process. | Methods, Risk of Bias Assessment, p. 6 (QUIPS; most-severe-domain decision rule) |
| Effect measures | 12 | Specify for each outcome the effect measure(s) (e.g. risk ratio, mean difference) used in the synthesis or presentation of results. | Methods, Statistical Analysis, p. 6 |
| Synthesis methods | 13a | Describe the processes used to decide which studies were eligible for each synthesis (e.g. tabulating the study intervention characteristics and comparing against the planned groups for each synthesis (item #5)). | Methods, Eligibility Criteria, p. 3–4; Methods, Statistical Analysis, p. 6 |
|  | 13b | Describe any methods required to prepare the data for presentation or synthesis, such as handling of missing summary statistics, or data conversions. | Methods, Data Extraction, p. 5; Methods, Statistical Analysis, p. 6 |
|  | 13c | Describe any methods used to tabulate or visually display results of individual studies and syntheses. | Methods, Statistical Analysis, p. 6; Tables 1–2, p. 7–10; Figures 2–3, p. 11–12 |
|  | 13d | Describe any methods used to synthesize results and provide a rationale for the choice(s). If meta-analysis was performed, describe the model(s), method(s) to identify the presence and extent of statistical heterogeneity, and software package(s) used. | Methods, Statistical Analysis, p. 6 (HKSJ with truncated variance correction; REML; R 4.5.2, meta package) |
|  | 13e | Describe any methods used to explore possible causes of heterogeneity among study results (e.g. subgroup analysis, meta-regression). | Methods, Statistical Analysis, p. 6 , no subgroup analysis or meta-regression was performed; the reason is stated there |
|  | 13f | Describe any sensitivity analyses conducted to assess robustness of the synthesized results. | Methods, Statistical Analysis, p. 6 |
| Reporting bias assessment | 14 | Describe any methods used to assess risk of bias due to missing results in a synthesis (arising from reporting biases). | Methods, Statistical Analysis, p. 6 , small-study effects not assessed (k = 3) |
| Certainty assessment | 15 | Describe any methods used to assess certainty (or confidence) in the body of evidence for an outcome. | Methods, Certainty of Evidence, p. 6 (GRADE adapted for prognostic-factor reviews; added at revision) |
| **RESULTS** | | |  |
| Study selection | 16a | Describe the results of the search and selection process, from the number of records identified in the search to the number of studies included in the review, ideally using a flow diagram. | Results, first paragraph, p. 7; Figure 1 (PRISMA flow diagram), p. 5 |
|  | 16b | Cite studies that might appear to meet the inclusion criteria, but which were excluded, and explain why they were excluded. | Results, first paragraph, p. 7 (the four studies excluded on re-verification, each with its reason); Methods, Study Selection, p. 4; full-text exclusions cited with itemized reasons in Supplementary material S2 |
| Study characteristics | 17 | Cite each included study and present its characteristics. | Table 1, p. 7; Results, study characteristics paragraph, p. 8; Table 2, p. 8–10 |
| Risk of bias in studies | 18 | Present assessments of risk of bias for each included study. | Results, Risk of bias, p. 13–14; Figure 4 (QUIPS traffic light), p. 13 |
| Results of individual studies | 19 | For all outcomes, present, for each study: (a) summary statistics for each group (where appropriate) and (b) an effect estimate and its precision (e.g. confidence/credible interval), ideally using structured tables or plots. | Table 2, p. 8–10; Figure 2, p. 11; Figure 3, p. 12 |
| Results of syntheses | 20a | For each synthesis, briefly summarise the characteristics and risk of bias among contributing studies. | Results, study characteristics paragraph, p. 8; Results, Risk of bias, p. 13–14 (including the weight carried by the study at high risk) |
|  | 20b | Present results of all statistical syntheses conducted. If meta-analysis was done, present for each the summary estimate and its precision (e.g. confidence/credible interval) and measures of statistical heterogeneity. If comparing groups, describe the direction of the effect. | Results, overall survival paragraph, p. 11; Results, progression-free survival paragraph, p. 12; Figures 2–3, p. 11–12 |
|  | 20c | Present results of all investigations of possible causes of heterogeneity among study results. | Methods, Statistical Analysis, p. 6; Results, p. 11–12 , I² = 0% for both outcomes and no investigation of heterogeneity was feasible at k = 3 |
|  | 20d | Present results of all sensitivity analyses conducted to assess the robustness of the synthesized results. | Results, overall survival paragraph, p. 11 (two substitution sensitivity analyses); Results, leave-one-out paragraph, p. 14; Figure 5, p. 15 |
| Reporting biases | 21 | Present assessments of risk of bias due to missing results (arising from reporting biases) for each synthesis assessed. | Methods, Statistical Analysis, p. 6; Results, p. 12 , not assessed, fewer than ten studies |
| Certainty of evidence | 22 | Present assessments of certainty (or confidence) in the body of evidence for each outcome assessed. | Results, Certainty of evidence, p. 14, including the justification for the one-level risk-of-bias downgrade (added at revision); Table 3, p. 10–11 |
| **DISCUSSION** | | |  |
| Discussion | 23a | Provide a general interpretation of the results in the context of other evidence. | Discussion, p. 15–16 |
|  | 23b | Discuss any limitations of the evidence included in the review. | Discussion, limitations paragraph, p. 17 |
|  | 23c | Discuss any limitations of the review processes used. | Discussion, search limitations paragraph and exposure-definition paragraph, p. 17 |
|  | 23d | Discuss implications of the results for practice, policy, and future research. | Discussion, p. 16–17; Conclusion, p. 17 |
| **OTHER INFORMATION** | | |  |
| Registration and protocol | 24a | Provide registration information for the review, including register name and registration number, or state that the review was not registered. | Methods, Study Design and Reporting, p. 3 |
|  | 24b | Indicate where the review protocol can be accessed, or state that a protocol was not prepared. | Methods, Study Design and Reporting, p. 3 |
|  | 24c | Describe and explain any amendments to information provided at registration or in the protocol. | Methods, Protocol Deviations, p. 3 |
| Support | 25 | Describe sources of financial or non-financial support for the review, and the role of the funders or sponsors in the review. | Funding, p. 18 |
| Competing interests | 26 | Declare any competing interests of review authors. | Conflict of Interest, p. 17 |
| Availability of data, code and other materials | 27 | Report which of the following are publicly available and where they can be found: template data collection forms; data extracted from included studies; data used for all analyses; analytic code; any other materials used in the review. | Data Availability Statement, p. 18 — OSF deposit 10.17605/OSF.IO/HUZJE (extraction sheet, per-study provenance, risk-of-bias judgements, analysis script); |

*From:*  Page MJ, McKenzie JE, Bossuyt PM, Boutron I, Hoffmann TC, Mulrow CD, et al. The PRISMA 2020 statement: an updated guideline for reporting systematic reviews. BMJ 2021;372:n71. doi: 10.1136/bmj.n71. This work is licensed under CC BY 4.0. To view a copy of this license, visit <https://creativecommons.org/licenses/by/4.0/>
